# High-volume online haemodiafiltration treatment and outcome of end-stage renal disease patients: more than one mode

**DOI:** 10.1007/s11255-020-02489-9

**Published:** 2020-06-02

**Authors:** Helmut Schiffl

**Affiliations:** Department of Internal Medicine IV, University Hospital LMU Munich, Ziemssenstr. 1, 80336 Munich, Germany

**Keywords:** End-stage renal disease, Haemodiafiltration, Expanded haemodialysis, Survival

## Abstract

The reduction of the dismally high mortality of current end-stage renal disease patients maintained on conventional standard haemodialysis (HD) remains an unmet medical need. Online haemodiafiltration (HDF) modes with various sites of fluid substitution (post-, pre-, mixed- and mid-dilution) are increasingly used worldwide as promising alternatives to conventional HD. Large scale cohort studies, post hoc analyses of randomized trials, and individual participant meta-analyses suggest that post-dilution and pre-dilution, especially with high substitution volumes, improve outcomes compared with conventional standard HD. However, there is no definitive proof of a survival advantage of HDF over standard HD. The different modes of high-volume HDF should be considered a therapeutic platform allowing to personalize and tailor routine HDF treatment. The selection of the HDF mode should be made according to individual patient characteristics. Utilizing high retention onset membranes, expanded haemodialysis (HDx) can achieve the same solute removal performance as HDF. Subgroups of high-volume OL-HDF patients could benefit from HDx. Ongoing and future trials should provide definitive proof for the superiority of high-volume OL-HDF over conventional HD or HDx to give guidance for the most favourable mode of dialytic therapy for clinical use.

## Introduction

Patients with end-stage renal disease (ESRD) maintained on dialysis have a shortened life expectancy and impaired quality of life compared to their peers without kidney disease, despite improvements in medical treatment. Death due to cardiovascular diseases—including arrhythmias, cardiac arrest, congestive heart failure, acute myocardial infarction and atherosclerotic heart disease is the largest known category of cause-specific mortality in dialysis patients. There is no convincing evidence of better survival with one type of dialysis (conventional haemodialysis) compared to another (peritoneal dialysis) [1].

Online haemodiafiltration (OL-HDF) represents the most technologically advanced convective form of blood purification and has the potential to improve outcomes in ESRD patients. OL-HDF is defined as a combination of diffuse and convective solute transport using a high-flux membrane with an effective ultrafiltration rate of at least 20% of the blood flow rate in combination with the use of an online-generated sterile and non-pyrogenic solution for fluid substitution [2].

The technological advances of OL-HDF confer clinical advantages over standard conventional HD. Plausible biological mechanisms underlying these effects are: (a) an increased removal rate of higher molecular weight solutes and some protein-bound uraemic compounds translates into a sustained lower residual uraemic syndrome; (b) better biocompatibility due to the combined use of biocompatible membranes and ultrapure/sterile fluids results in a reduction in systemic inflammatory response and (c) a favourable impact of OL-HDF on intra-treatment hypotensive episodes due to multiple mechanisms including a higher sodium mass transfer and mode-specific thermal effects (Table 1) [3, 4].

**Table 1:** Principles of high-volume OL-HDF

	High-volume OL-HDF dilution mode		
	Post	Pre	Mixed
Convective clearance (middle molecules)	+++	++	+++
Diffusive clearance (small solute)	+++	+	+++
Biocompatibility	+++	+++	+++

This literature review describes the content and quality of knowledge about clinical benefits associated with high-volume OL-HDF modes in ESRD patients and gives a critical appraisal of the clinical relevance of the reported data.

## Modes of online HDF

Convective therapies coming under the umbrella of high-efficiency HDF involve larger volumes of infusion than other convective therapies. Various modes of OL-HDF, differing in the site of replacement fluid infusion are in use. However, only four modalities of OL-HDF are currently used in different countries: post-dilution HDF, pre-dilution HDF, mixed-dilution HDF and mid-dilution HDF (Table 2) [2]. The use of OL-HDF is increasing worldwide, but variable due to patient and centre specific characteristics [3].

**Table 2:** Modes of high-volume online haemodiafiltration (OL-HDF)

Mode of HDF	Replacement volumes
Post-dilution OL-HDF	Ultrafiltration followed by infusion of sterile replacement fluid
Pre-dilution OL-HDF	Infusion of sterile replacement fluid followed by ultrafiltration
Mixed-dilution OL-HDF	Infusion of sterile replacement fluid before and after ultrafiltration
Mid-dilution OL-HDF	Infusion of sterile replacement fluid at the midpoint of ultrafiltration

High-volume high-efficiency post-dilution offers—at least in theory—the most effective removal of middle molecule solutes. This mode is widely spread in Europe and other parts of the world. However, high-efficiency post-dilution OL-HDF is often limited by inappropriately high haemoglobin levels due to correction of renal anaemia and increased protein concentration which could result in a critically increased trans-membrane pressure, membrane fouling and consequently to impaired removal of solutes. Haemoconcentration generally limits the rate of ultrafiltration to a filtration fraction of 20–30% of the blood flow rate in post-dilution HDF [2].

Ultrafiltration rate is not limited by the blood flow rate in-pre-dilution HDF. However, pre-dilution HDF reduces the efficiency of both the diffusion and convective components of solute removal. To match the efficiency of post-dilution OL-HDF, the inherent dilution should be compensated for by increasing the substitution volume by a factor of two in the pre-dilution mode [2–5]. In mixed-dilution OL-HDF, the substitution fluid is administered simultaneously in pre-dilution and post-dilution, regulated by the trans-membrane pressure feedback, which automatically adjusts and controls the infusion ratio between pre-dilution and post-dilution, as well as the total infusion volume as a sum of both [6]. Mixed-dilution HDF ensures more favourable blood rheology and membrane permeability than the post-dilution mode and allows the total infusion to be increased and the convective solute removal to be forced beyond the limits of post-dilution HDF [6]. Mixed-dilution OL-HDF results in significantly higher convective removal of small and middle molecular uraemic toxins than pre-dilution OL-HDF, while maintaining the optimal pressure conditions within the dialyzer better than pre- or post-dilution HDF [2–6].

Successful high-volume post-dilution HDF depends on high blood flow rates (≥ 350 mL/min), an excellent vascular excess (arteriovenous fistula blood flow rates ≥ 600 mL/min), an ability to achieve adequate anticoagulation throughout the HDF session, and the absence of any clinical disorder that increases blood viscosity (cryoglobulinemia, gammopathies and polycythaemia) (Table 3). Central venous catheters should not be seen as an obstacle for post-dilution OL-HDF. However, in a multi-centre study only one third of the patients with catheters achieved the minimum replacement volume target of 21 L [7]. Accordingly, the duration of HDF sessions must be increased at least for 30–60 min in ESRD patients with blood flow rates of 250–300 mL/min. Consequently, patients with vascular access problems who refuse to extent HDF sessions are not suitable for high-volume HDF.

**Table 3:** Performance of high-volume OL-HDF modes

Technical requirements for high-volume HDF
Certified online HDF machines with ultrafiltration control
Haemodialyzer (high-flux membrane (1.6–2.2 m^2^), ultrafiltration coefficient > 20 mL/h/mmHg/m^2^, sieving coefficient > 0.6 for β2-microglobin)
Online production of sterile and non-pyrogenic substitution fluid
Adequate prescription of high-volume OL-HDF
Vascular access, arteriovenous fistula or graft, tunnelled central vein catheter
Blood flow rates: post-dilution or mixed-dilution 350–450 mL/min, pre-dilution OL-HDF 200–250 mL/min
Dialysate flow rate > 500 mL/min
Convection volume: post-dilution > 23 L per treatment; pre-dilution 50 L per treatment; mixed dilution > 35 L per treatment
Dialysate composition according the patients’ needs
Anticoagulation (unfractionated or low molecular heparin)
Duration > 4 h three times per week
Regular quality assessments, adherence to hygienic standards, education of the staff

One of the marked characteristics of management of ESRD patients in Japan, is the widespread use of pre-dilution HDF. More than 95% of patients maintained on regular HDF are treated with this mode of OL-HDF. The main clinical reason for the exclusive use of pre-dilution OL-HDF is the low blood flow rates in Japanese ESRD patients [8].

Regarding the issue of safety of high-volume online HDF, there seems to be no indication that infusion of large amounts of online produced fluid causes a chronic inflammatory state, provided that strict hygienic standards are applied. Penne et al. analysed more than 11,000 HDF treatment sessions and demonstrated that production of high-quality substitution fluid is possible over a prolonged period of time [9]. Furthermore, the use of high-volume post-dilution HDF is not associated with a risk of fluid imbalance [3].

Whether the use of modern dialyzer membranes exposes the patient to a greater risk of loss of albumin needs to be further analysed by controlled clinical trials. Compared to traditional high-flux membranes, new membranes, referred to as protein leaking membranes, super-flux or high-performance membranes, improve the clearance of proteins in the middle to high weight range and highly protein-bound uraemic toxins at the cost of increased albumin losses. When prescribing post-dilution OL-HDF the choice of the membrane should be made with caution. A recently published case report illustrates that the use of a steam sterilized polyphenylene membrane during post-dilution OL-HDF can lead to significant albumin loss into the dialysate and severe hypoalbuminaemia in an individual patient [10].

## Clinical benefits of various modes of high-volume OL-HDF

### Post-dilution OL-HDF

The expectation that post-dilution OL-HDF may be beneficial originates primarily from innumerable cohort studies but only 4 randomized controlled trials (RCTs). The results of these studies suggested, albeit not consistently, that post-dilution OL-HDF is associated with less morbidity and lower mortality than conventional HD.

The observed clinical advantages provided by high-volume HDF techniques, particularly post-dilution OL-HDF encompass (a) better intra-treatment haemodynamic stability; (b) less inflammation induced malnutrition, atherosclerosis and erythropoietin resistance; (c) fewer cases of dialysis related amyloidosis and uraemic polyneuropathy; (d) improved derangement in calcium-phosphate homeostasis and less vascular calcification; (e) higher treatment dose by better preservation of residual renal function and higher KT/V; (f) improved quality of life with less insomnia, restless leg syndrome, itching or joint pain [4, 8, 11].

There is some controversy on the potential effects of post-dilution OL-HDF for the reduction of mortality risk. Well-performed, large scale cohort studies, post hoc analysis of three large RCTs and meta-analyses of four RCTs suggest that post-dilution OL-HDF could improve patient mortality. Most randomized controlled trials were not designed to study the effect of effective dose. Nevertheless, reported patient survival was greater for those receiving greater convective volume exchange. Reduced mortality (by 22%) with post-dilution HDF was observed with pooled analyses of patient data of the four trials, due to a reduction of cardiovascular events (by 31%), with authors estimating the prevention of one cardiovascular death for every 75 patient years of treatment [12, 13]. Current recommendations of the adequate dose of regular high-volume post-dilution HDF are a convection volume ≥ 23 L/session administered thrice weekly. The ongoing CONVINCE study, a large, international, multi-centre, randomized, controlled trial is set out to prove the superiority of high-dose post-dilution OL-HDF as compared to high-flux standard HD in terms of morbidity, mortality, and health-related quality of life [14].

### Pre-dilution OL-HDF

Retrospective small observational studies suggest that pre-dilution OL-HDF may ameliorate various uraemic symptoms, such as loss of appetite, resulting in an improved nutritional status and preserved muscle volume, relief of shoulder pain and lower prevalence of dialysis related amyloidosis, itching, insomnia and restless legs syndrome [15, 16]. A study conducted by the Japanese Society for Haemodiafiltration (JSHDF) showed the non-inferiority of pre-dilution OL-HDF with regard to intradialytic haemodynamic stability compared with the post-dilution mode [8]. Ohatake and co-workers prospectively compared atherosclerotic and cardiac functional surrogate markers in patients randomized either to high-volume pre-dilution HDF or conventional HD. This small RCT suggested a cardiovascular protective effects of pre-dilution OL-HDF [17].

The association of improved survival with pre-dilution OL-HDF has been recently evaluated by retrospective analyses. A small Greek study noted a survival benefit of pre-dilution OL-HDF over peritoneal dialysis 2–2.5 years after treatment initiation [18]. The study conducted by the Japanese Society for Dialysis Therapy (JSDT) found a clear survival benefit for all-cause mortality and cardiovascular mortality of pre-dilution OL-HDF ≥ 40 L/session. No survival advantage was observed with a substitution volume < 40 L/session [8]. Using the Japanese Society for Dialysis Therapy Renal Data Registry database Kikuchi et al. created a propensity-matched cohort of 5000 pairs of patients treated with conventional HD or pre-dilution OL-HDF [19]. Pre-dilution OL-HDF was associated with improved survival compared to conventional HD (HR = 0.83) with a trend towards improved cardiovascular survival. Patients treated with high substitution volumes (≥ 40 L/session) had improved all-cause and cardiovascular survival compared to those treated with lower substitution volumes (< 40 L/session). The optimal substitution volume with improved survival was estimated to be 50.6 L/session.

### High-volume mixed-dilution HDF

Preliminary results of its clinical application indicate that trans-membrane pressure (TMP)- modulated mixed-dilution OL-HDF could be one of the most powerful strategies to prevent or delay the occurrence of some long-term dialysis complications and to promote improved survival of ESRD patients [6]. However, this mode of OL-HDF is not widely used and available data are preliminary.

A recent retrospective multi-centre 1-year study aimed to explore whether mixed-dilution OL-HDF improves anaemia management compared to traditional post-dilution HDF. 174 matched prevalent ESRD patients were included. 87 patients had received mixed-dilution HDF (mean substitution volume 38 ± 5 L/session) and 87 patients were on post-dilution HDF (mean substitution volume 24 ± 4 L/session. The result of this explorative study suggested that patients on mixed-dilution HDF may have a greater clinical benefit in terms of anaemia control than patients treated with post-dilution OL-HDF [20]. A small Irish study compared conventional HD for 6 months, post-dilution OL-HDF for 6 months and mixed-dilution OL-HDF for 6 months. The author found that post-dilution HDF and mixed-dilution HDF had a superior quality of life (higher physical and mental components scores) than conventional HD [21].

## Clinical indications for high-volume OL-HDF modes

High-volume OL-HDF is indicated for all ESRD patients (incident or prevalent) and opens an avenue to personalized treatment of ESRD (Table 4). High-volume OL-HDF represents the preferred dialysis modality for paediatric patients to prevent growth retardation. Young ESRD patients facing need for long-term dialysis are candidates for high-volume OL-HDF to prevent long-term dialysis related morbidity and mortality. Patient with persistent symptoms of uraemia or patients with intolerance of haemodialysis are suitable for high-volume OL-HDF. Enhanced removal of uraemic molecules and better haemodynamic stability during treatment may be associated with symptom relief and improved quality of life. However, ESRD patients with certain haematologic disorders or difficult vascular access as well as patients nonadherent to fluid restriction or not willing to prolong HDF sessions are not suited for high-volume post-dilution OL-HDF but can be treated with pre-dilution or mixed-dilution high-volume OL-HDF.

**Table 4:** Selection criteria for mode of HDF

General indications
There are no contraindications for high-volume OL-HDF
Patient age related indications
Children/adolescents
Young age and need for long-term dialysis treatment
Persistent symptoms of uraemia
Dialysis-related amyloidosis
Uraemic neuropathy (restless legs syndrome, sleep disturbances)
Uraemic pruritus
Prevention of skin hyperpigmentation
Intolerance of haemodialysis sessions
Nausea, vomiting
Cramps
Headache
Post-dialysis fatigue
Frequent symptomatic episodes of intradialytic hypotension
Specific indications
Specific diseases (high blood viscosity, cryoglobulinaemia, gammopathies, high haematocrit)
Poor vascular access
Slightly built body
Nonadherence (excessive interdialytic weight gain, patients unwilling to accept prolonged treatment sessions)

As claimed by Tattersall [2], Canaud [3], Ronco [4], Masakane [8]

## Expanded haemodialysis (HDx): an emerging alternative to high-volume OL-HDF?

HDx has emerged as a potential breakthrough of haemodialysis techniques [22, 23]. Medium cut-off (MCO) membranes are a new class of dialyzer membrane with a cut-off close to the molecular weight of albumin. They enhance the removal of molecules traditionally retained with current dialysis membranes and techniques [22]. These membranes have a very high retention onset, while presenting negligible albumin loss (< 3.5 g per session), allowing high clearances of solutes as measured by the removal ratio (RR) or the global removal score in a wide spectrum of molecular weights [24]. The pilot study by Maduell et al. compared one MCO dialyzers with eight haemodiafiltration dialyzers [25]. No significant differences in the RRs of small and middle molecules, and the global removal scores were observed between MCO and HDF dialyzers. MCO dialyzer membranes even may allow the removal of larger uraemic toxins not previously targeted by current maintenance HD techniques [26, 27]. The MCO dialyzer membranes offer effective retention of bacterial products from conventional dialysis fluid without requiring an ultrapure water supply or other HDF infrastructure [28].

The MCO HDx may be a valuable option for ESRD patients who don't reach the large blood flow rates required for high-volume OL-HDF or when HDF is not available. Patients treated with high-volume OL-HDF could also benefit from HDx whenever HDF needs to be suspended.

Limited studies have demonstrated potential clinical benefits of MCO membranes in reducing cardiovascular risk by reduction of vascular calcification [29] and by decreasing the inflammatory allostatic load associated with retention of middle molecular weight uremic toxins [30]. With the potential of better removal of larger middle molecules, HDX may improve pruritus and restless legs syndrome [31]. Further clinical studies are needed to assess whether the HDx may improve the outcome of ESRD patients.

## Conclusions

The current uncertainty of the optimal dialysis strategy for ESRD patients results in a heterogenous delivery of care, which is not driven by the best evidence but by empiricism and local centre performance. Definitive proof for a survival benefit of high-volume OL-HDF is still needed. Currently, the mode of OL-HDF should be established according to characteristics and needs of individual patients. It would be worthwhile if further randomized trials prove the superiority of high-volume HDF over conventional or HDx.
